# Critical Review of the State-of-the-Art on Lumbar Percutaneous Cement Discoplasty

**DOI:** 10.3389/fsurg.2022.902831

**Published:** 2022-05-10

**Authors:** Chloé Techens, Peter Endre Eltes, Aron Lazary, Luca Cristofolini

**Affiliations:** ^1^ Department of Industrial Engineering, Alma Mater Studiorum - Università di Bologna, Bologna, Italy; ^2^ In silico Biomechanics Laboratory, National Center for Spinal Disorders, Budapest, Hungary; ^3^ Department of Orthopaedics, Department of Spine Surgery, Semmelweis University, Budapest, Hungary

**Keywords:** percutaneous cement discoplasty, minimally invasive spinal surgery, spine biomechanics, clinical outcome, systematic review

## Abstract

Interbody fusion is the gold standard surgery to treat lumbar disc degeneration disease but can be a high-risk procedure in elderly and polymorbid patients. Percutaneous Cement Discoplasty (PCD) is a minimally invasive technique developed to treat advanced stage of disc degeneration exhibiting a vacuum phenomenon. A patient-specific stand-alone spacer is created by filling the disc with polymethylmethacrylate cement, allowing to recover the disc height and improve the patient’s conditions. As it has recently been introduced in the lumbar spine, this review aims to present a transversal state-of-the-art of the surgery from its clinical practice and outcome to biomechanical and engineering topics. The literature was searched across multiple databases using predefined keywords over no limited period of time. Papers about vertebroplasty were excluded. Among 466 identified papers, the relevant ones included twelve clinical papers reporting the variations of the surgical technique, follow-up and complications, four papers reporting biomechanical *ex vivo* and numerical tests, and four letters related to published clinical papers. Papers presenting the operative practice are reported, as well as follow-ups up to four years. The papers found, consistently reported that PCD significantly improved the clinical status of the patients and maintained it after two years. Spine alignment was impacted by PCD: the sacral slope was significantly reduced, and disc height increased. The foramen opening correlated to the volume of injected cement. Substitutes to the acrylic cement exhibited better osteointegration and mechanical properties closer to bone tissue. Finally, limitations and risks of the surgery are discussed as well as potential improvements such as the development of new filling materials with better mechanical properties and biological integration or the investigation of the inner disc.

## Introduction

The ageing of the global population due to the increase of life expectancy directly increases the prevalence of spine disease and in particular degeneration of the lumbar Intervertebral Disc (IVD) (1). With time, the IVD water content decreases leading to tissue breakdown and to loss of disc height (2). Consequently, the foramen space between adjacent lumbar vertebrae is reduced, creating neural stenosis and inducing low back pain in some cases (3). In the most extreme degrees of disc degeneration, the nucleus is replaced by a vacuum phenomenon (VP), creating a large instability of the spine segment and extreme compression of the nerves (4).

Lumbar IVD degeneration treatments range from physiological exercises to surgical procedure. Depending on the stage of the disease, the invasiveness level of the treatment strategy varies. At an early stage, conservative management is prioritized. In this case, restorative, reconstructive or disc replacement strategies are applied: a review on this topic has recently been published (5). The most common surgical solution, with the longest follow up is interbody fusion, requiring insertion of a cage and bone graft combined to posterior fixations to restore the intervertebral height and stabilize the spine. Pain-relieving injections and molecular treatments such as cell, growth factor, and gene therapies (6) have been developed to handle early stages of the degenerative process. Reconstructive strategies include percutaneous techniques for decompression and biomaterial implantation (7). Finally, for advanced degeneration, total disc arthroplasty and particularly rigid fusion are favoured (8). This late surgical technique is a long surgical procedure requiring a general anaesthesia and a long recovery. It is also associated with high risks of bleeding and complications. Therefore, it can be contraindicated for elderly and polymorbid patients. For those unsuitable patients, the absence of efficient treatment led to the development of minimally invasive technique called Percutaneous Cement Discoplasty (PCD) (9).

PCD is dedicated to treat patients with advanced disc degeneration exhibiting a VP. The procedure consists in the injection of an acrylic cement within the disc to fully fill the cavity. The cement mass then acts as a stand-alone implant, restoring the disc height.

Historically, a similar technique has been implemented in the cervical spine as an alternative to interbody fusion cages for spine segment stabilization. Injection of bone cement in the disc was introduced in the Eighties by Roosen (10). The technique was then replicated *in vivo* (11, 12) and *in vitro* (13–15) to investigate the surgical outcome and biomechanical consequences of such a treatment on the cervical spine in comparison to spacer. It was found that acrylic cement stabilized the spine similarly to other cages (11, 12, 15), but showed a lower subsidence in adjacent vertebrae (13, 14).

Thus, PCD is considered as a promising technique for spinal repair. However, the knowledge around the surgery and its consequences on the lumbar spine is still under investigation. Papers have been recently published on several aspects of PCD, from clinical cohort papers to engineering papers on biomechanics and biomaterials.

This review aims to present the various research areas related to PCD to provide a clear view of the progresses and needs in this field. The review aimed to assess of the efficiency of this technique in terms of clinical outcome for the patient, but also in terms of objective parameters such as spinal behaviour and spine stability.

## Methods

### Search Strategy

This review includes papers of all types from articles to letters to the editor in peer-reviewed journals. No single study design was specified since the review aimed to collect all PCD-related publications. No time frame was defined although first publications mentioning PCD were published in 1982 and reporting lumbar PCD in 2015. Only peer-reviewed publications with an English version were considered.

The review established a state-of-the-art about PCD. Therefore, the inclusion criteria rather targeted the qualificatives of PCD to ensure both quantitative and qualitative papers to be retrieved. The review focused on surgical practices applied on the intervertebral discs of the thoracolumbar spine and consisting of injecting acrylic bone cement within a disc presenting a vacuum phenomenon. Papers about vertebroplasty were excluded as well as surgeries which fixed the spinal posterior elements.

**Figure 1:**
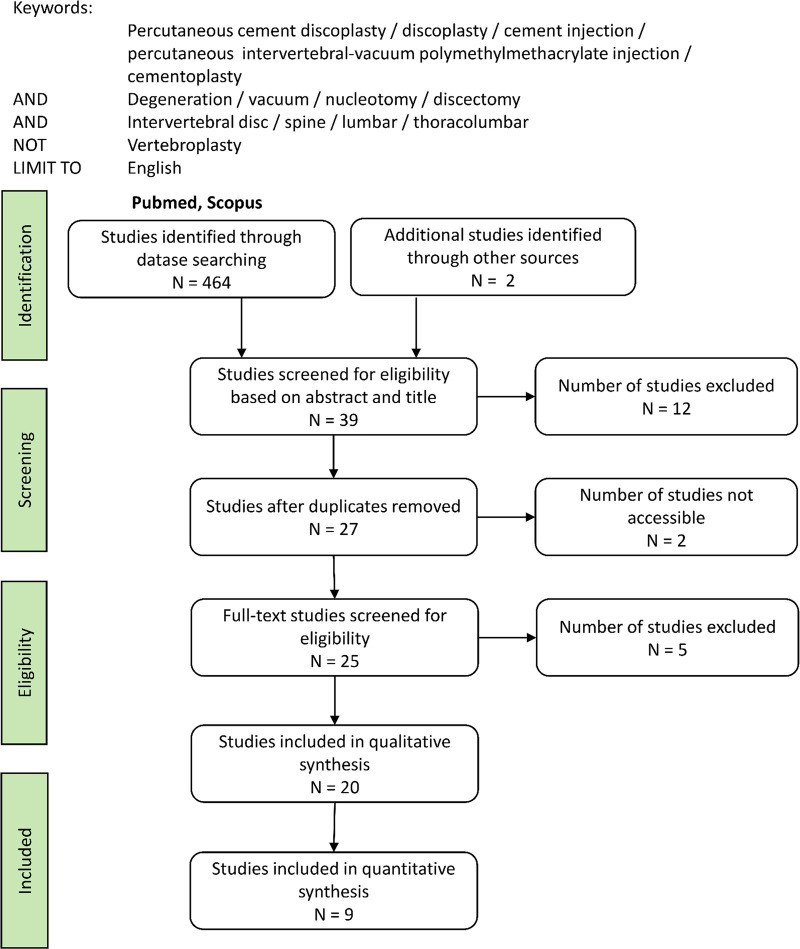
Workflow of the search strategy.

The search was performed on the electronic databases PubMed and Scopus. Additionally, the references of the screened papers were reviewed to search potential related studies (Figure 1).

The papers collected from the databases were checked for duplicates. A first screening was based on the titles and abstracts of the papers, to ensure that the papers indeed focused on the intended topic and was not picked erroneously. The final eligibility of the papers was based on the full text content to fully assess all criteria. This process was initially performed by one author, but the results were approved by the others. Among the eligible papers, separation was performed between qualitative papers assessing the characteristics of the surgery and its consequences, and papers including quantitative evaluation of PCD outcome.

### Data Collection Process

Qualitative and quantitative data were then extracted from the papers using a form established by the authors to assess the quality of the papers and their content. The variables sought in all papers were:

- Type of the study (cohort/retrospective/prospective/*in vitro*/numerical)
- Presence and clarity of the inclusion criteria of specimens/patients in the study
- Presence and clarity of the exclusion criteria of specimens/patients from the study
- Presence of comparison between groups of persons/patients undergoing two different treatments

As the review covered various types of papers from clinical to biomechanical papers, additional variables were investigated, most being suitable for the majority of the papers:

- Presence and duration of a follow-up
- Period of the study
- Number of persons in the cohort/specimens
- Inclusion/exclusion criteria for the persons/specimens
- Variables observed and corresponding parameters measured
- Frequency of measurement
- Nature of the parameters’ outcome (index, scale, cases)
- Presentation of the operative technique
- Monitoring of the surgery
- Surgical approach chosen
- Use of preliminary medium to assess the volume of cement to inject
- Volume of cement injected
- Duration of the surgery
- Discharge of the patients
- Post-operative treatment/recommendation
- Presence of case presentation
- Complications/limitations

In particular, the review investigated patient outcomes, the operative technique, and potential risks induced by PCD on the spine depending on the type of article collected. For that, a particular interest was brought to:

- Patient self-reported pain, mobility, etc.
- Patient mobility assessed objectively
- Spinal alignment
- Mechanical behaviour of spine
- Disc height/foramen size changes
- Complications/risks

Risk of bias was also verified both at the study level (related to funding for instance) and at the outcome levels. Among the practices recommended to decrease the risk of bias, one can mention the use of an independent observer or a double-blind, the repeatability of measurements, the reproducibility of the measurements by two operators. Conversely, self-reporting of the patient pain would represent subjective results although it is a crucial tool in clinics. This review did not aim to hierarchize some results over others, but to make the reader aware of potential weaknesses and limitations of the available data. Each field of research has its own tools which fill the field needs and complete each other.

Qualitative data were reported, gathering into groups the papers presenting similar values. For quantitative parameters, the mean values reported in each paper were compared using the same scale. To quantify patient’s quality of life and pain, Oswestry Disability Index (ODI) and Visual Analogue Scale (VAS) scores are reported using the standard scale from 0 to 100. Spine alignment and stability will be quantified by anatomical parameters in terms of angles and distances.

## Results

### Results of the Literature Search Process

The search on PubMed and Scopus with the keywords stated above (Figure 1) resulted in respectively 32 and 432 papers of all types. In addition, a study conducted by our group and currently in submission was included to the published papers. Reviewing the references of these papers, one more publication was included in the panel. The first screening of the abstracts and titles provided 27 eligible papers. The full-text reading established that 20 publications were qualified for this review on PCD, all written after 2015. Among them, 15 were identified as journal articles covering both clinical and biomechanical investigations, and 5 as letters to the editors commenting some published articles.

The articles found included four prospective studies (9, 16–18), one case study (19), two diagnostic studies (20, 21), five retrospective studies (22–26), four biomechanical studies whose only three published (27–29). The four remaining publications were correspondence to the Editor articulated around two distinct conversations. Following the case study presented by Sola et al., a first letter to the editor was written by Wang et al. to require more details about the operative technique and the outcome (30). The content of the answers from Camino-Willhuber and Sola was published in another letter (31). Additionally, Lazary commented on Sola et al.’s case study, questioning the need of intraoperative neurophysiological monitoring (32). Camino-Willhuber et al. explained their use of the technique with regards to their own surgical experience (33).

Except for the case study, the diagnostic studies, and the letters, all papers provided quantitative data tackling the patient outcome and/or biomechanical parameters. All papers acknowledged their risks of bias and tried to mitigate them.

One must note that the term Percutaneous Cement Discoplasty was not universally used in the literature. Yamada et al. reported the surgical technique in their two papers under the name percutaneous intervertebral-vacuum polymethylmethacrylate injection (PIPI) while Tian et al. used the term percutaneous disc cementoplasty (PDCP). In this review, the surgical technique is named after the most common term: percutaneous cement discoplasty (PCD, *n* = 16 hits in total) rather than PIPI (*n* = 2 hits) or PDCP (*n* = 2 hits).

Among the recorded 20 publications, eight papers included a follow-up involving the recruitment of human participants. Yamada et al. compared groups undergoing PCD to other treatments, whereas the others focused on a preop/postop comparison. For *in vivo* papers, the selection process of the participants was explicitly detailed in the text at minimum, with additional scheme to summarize in Yamada et al. and Kiss et al. papers.

This review gathers all publications linked to PCD, whether they covered the patient outcome or the operative technique. Data collected *in vivo* and *in vitro* are presented separately below. A summary table of the literature results is available in a Figshare file (https://doi.org/10.6084/m9.figshare.19375604).

### Operative Technique

#### Chronological Presentation of PCD Technique

Sixteen publications tackled PCD applied to patients, from the surgical planning to the operative technique itself, and covering the patient outcome. Historically, cement injection in the IVD was primarily introduced to stabilize the cervical spine (10, 15). In 2015, Varga et al. presented the operative technique applied for the first time to the lumbar spine (9), followed in 2018 by Sola et al. (19). Two papers presented case studies (9, 19). Camino-Willhuber et al. focused on the development of a methodology to fine-tune the diagnosis of cases requiring PCD as a treatment (20). Eltes et al*.* developed a methodology to quantitatively evaluate the impact of the surgery on patient anatomy using medical imaging (21). Finally, ten papers included a follow-up of the patients (9, 16–18, 21–26). While Kiss et al. and Varga et al. (9, 16) investigated PCD as treatment of disc degeneration to restore vertical stability, Yamada et al*.* applied PCD to specifically treat scoliosis resulting from disc degeneration (17, 18). The paper compared the clinical outcomes of two groups: patients treated with PCD, and patients treated with physiotherapy. Camino-Willhuber et al*.* addressed the matter by comparing the treatment outcome in patients with and without degenerative scoliosis (22). Another paper by Camino-Willhuber et al*.* compared the PCD outcome between three groups of patients depending on their previous spine surgical history at the treated level (24). Finally, Tian et al. reported using PCD after percutaneous lumbar discectomy to treat lumbar disc herniation in two papers (25, 26). One must note that these papers differ in terms of indications of PCD: contrary to the original paper recommendation (9), PCD aimed there to treat a spinal condition unrelated to disc degeneration disease.

#### Surgical Planning

All authors except Tian et al. defined the same indications for surgery as introduced by Varga et al. As a minimally invasive surgery, PCD is mainly intended to treat patients not suitable for an open surgery. Eligible patients suffer from a Disc Degeneration Disease in an advanced stage (Pfirrmann’s grade V) resulting into a VP due to the disappearance of nucleus pulposus. Evidence of foraminal stenosis directly inducing back pain is also an indication, and specifically when pain increases with standing activity and is relieved after resting (https://doi.org/10.6084/m9.figshare.19375604).

Pfirrmann’s scale evaluates the intervertebral disc degeneration stage; however PCD principally depends on VP and the surrounding tissue state. For this reason, surgery planning was refined to identify patients having the most suitable pathological condition of the disc and the endplates (20). A new classification of VP, established from Computed Tomography scans, identified four levels of VP based on the rate intervertebral vacuum/disc tissue and two sub-levels depending on the presence of subchondral stenosis. Camino-Willhuber et al*.* suggested that PCD should be only recommended for partial or complete VP, to reduce the risks of disc protrusions during acrylic cement injection. Additionally, the presence of subchondral stenosis would limit risks of adjacent fractures, in particular in osteoporotic patients.

Some contraindications were presented by Sola (19):

- Severe osteoporosis could jeopardize the integrity of the vertebral bodies after the surgery. Following Wang’s letter to the editor (30), Camino-Willhuber and Sola specified that no direct measure of lumbar osteoporosis was used as a threshold to discriminate patients suitable for PCD (31). However, patients with a T-score lower than −2.5 at the hip, or history of bone fracture were referred to endocrinologist for anti-osteoporosis treatment. In their papers, Yamada et al*.* defined a bone density threshold of 70% of the young adult mean measured by dual-energy x-ray absorptiometry, under which the surgery was not recommended.
- Severe deformity of the spine would exclude patients from receiving PCD. Indeed, although this surgery demonstrated a stabilizing effect on the spine in case of degenerative scoliosis, PCD does not aim to correct severe deformities (31).
- Evidence of tumours, metastases, or infections at the corresponding spine levels.
- Obesity is a limiting factor because it reduces the quality of the fluoroscopy monitoring required during the surgery

Tian et al*.* presented a different use of PCD (25, 26)*.* In their papers, the combination of percutaneous discectomy and PCD was studied as a treatment to lumbar herniation with endplate osteochondritis. Because percutaneous discectomy alone cannot treat the late condition, PCD was performed as a second step of the surgical treatment. Hence, the recruitment of patients in these studies differed from the criteria above. Eligible patients demonstrated neurological signs related to disc migration with endplate osteochondritis, contained disc protrusion with Modic type I changes of the endplate bone marrow, had no history of surgery at the disc level, and were above 60 years old. Patients were also included after at least 6 weeks of unsuccessful conservative treatment. Similarly, patients were excluded in case of spinal nervous canal stenosis (grades 2 and 3 of Lee et al. (34) and Bartynski and Lin (35) classifications), sequestered disc below or above the centre of the pedicle of the lower vertebral body, calcification of longitudinal ligaments, comorbidities such as cardiovascular disease, diabetes, infection, spinal tumour, or fracture, untreatable coagulopathy, and allergy to polymethyl methacrylate (PMMA).

#### Surgical Procedure

PCD is a minimally invasive surgery; its operative technique is described by two papers. A radiopaque bone cement was injected to fill the vacuum using an extra-pedicular approach through the Kambin’s triangle (9, 16, 32). Yamada et al*.* prioritized a transpedicular approach for the injection, while Sola et al. also recommended an entrance parallel to the superior lateral pedicle edge except for L5-sacrum level (33). Tian et al. favoured a posterolateral puncture of the disc. Wang et al*.* confronted the difference of approaches used by Varga and Sola et al*.*, questioning the key factor allowing a homogeneous cement distribution and avoiding leakages (30). Camino-Willhuber and Sola recommended inserting the cannula between middle and anterior third of intervertebral. Stopping injection when bone cement reaches the posterior vertebral wall would prevent leakages (31). If Varga et al*.* recommended local anaesthesia, what did Tian et al., PCD can be conducted under general anaesthesia as reported by Camino-Willhuber, Kiss and Yamada et al*.* (16–18, 22, 24). For all papers, the volume of injected cement varied between 3–10 mL depending on the patient and spine level, since cement must entirely fill the vacuum. Because the vacuum was artificially created by percutaneous discectomy, cement volume reported by Tian et al. was slightly inferior (25, 26). The surgery was always performed under fluoroscopic monitoring for a better guidance of the injection and to prevent cement leakage in the neural canals. In addition, Sola et al. recommended the systematic use of intraoperative neurophysiological monitoring during the whole surgery. Lazary argued that risk of nerve root injuries is minimal as long as the surgical rules is followed, and fluoroscopy guidance used (32). Considering the increased cost and duration of PCD procedure caused by neuro-monitoring, its systematic use would not be encouraged. Besides, in the experience of Lazary’s group, none of the treated patients suffered from nerve root injuries. Camino-Willhuber explained that neuro-monitoring is specially recommended for the Kambin’s triangle approach which presents more risks of nerve root injuries, in particular in case of deformity (33). Neuro-monitoring, installed during anaesthesia induction, allowed to prevent radicular irritation by changing the cannula entry point in their practice without increasing surgical length. A study on the utility of intraoperative neurophysiological monitoring during PCD reported a sensitivity of 82% and specificity of 99% (23). Before cement injection was introduced, one paper used a medium injected in the disc to assess the volume of required cement (17). The surgery duration varied between papers, depending on the number of treated levels, from about 25 min for one level PCD to more than 1 h for five level PCD. Camino-Willhuber et al. demonstrated that PCD associated with decompression surgery in cases with spinal stenosis, also provided promising outcome to treat the patients (24). Decompression surgery could also be directly indicated from the results of the intraoperative neurophysiological monitoring in case of leakage (33).

#### Complications and Postoperative Recommendations

Kiss et al. and Yamada et al*.* reported cement leakages in 4% of the surgeries (respectively 3/63 patients and 3/80) which were treated by decompression surgery (16, 18). In the first paper, all leakages, located in the foramens, caused severe leg pain, and were treated by foraminal decompression during a revision surgery. In the second paper, one leakage was localized in the intervertebral foramen and induced a radicular pain which was treated with anti-inflammatory analgesics. In their papers, Tian et al. reported 1/7 and 2/16 leakages inducing slight pain but the symptoms disappeared within 24 h without treatment (25, 26). Because of the reduced capacity of the disc after PCD to homogeneously transmit the vertical stress at the endplate levels, Wang et al. shared concerns about the increase of fracture risk (30). In their answer, Camino-Willhuber and Sola reported one fracture over 131 treated discs. They explained that fractures were prevented by the degeneration of the endplates which resulted in subchondral sclerosis. No endplate fracture nor cement dislodgement was reported by Yamada et al. (17). One deep infection and one fracture of the adjacent vertebral body were later reported by Camino-Willhuber et al. along with two cases of leakage in the foramen, one disc extrusion and one unexplained pain (22). Overall, in their last paper complications were reported to affect 16% patients, with only 5.7% (9/156) requiring a second operation (24). Cement leakage accounted for 3.2% and vertebral fracture for only 0.6%.

Patients were usually discharged within 3 days, and were encouraged to stand and walk as soon as possible (22). When PCD was associated with lumbar discectomy to treat herniation, the hospitalization lasted about 7 days (25, 26). In the case of the treatment of lumbar degenerative scoliosis, a brace was worn by patients for two months (17). Camino-Willhuber’s group did not recommend a brace postoperatively, since patients undergoing PCD did not have risky activities. The only recommendation was to avoid excessive flexion/extension movements and avoid lifting more than 10 kg (31).

### Clinical outcome

Among the nine papers including a follow-up, the shortest follow-ups lasted six months (9, 16). Camino-Willhuber et al. presented a 12 months follow-up (22) and a second study of 24 months follow-up (24). Tian et al. presented a 12 months follow-up (25) and a second study with an averaged follow-up of 39 months (26). Yamada et al. first paper measured patient outcome for 24 months (17), the second study based on the same cohort lasted about 63.7 ± 32.4 months (mean ± SD) (18). Periods over which the recruitment and the follow-up of patients was performed widely varied between papers. All details of the follow-ups are summarized in the Figshare file (https://doi.org/10.6084/m9.figshare.19375604).

#### Selection of Patients

**Figure 2:**
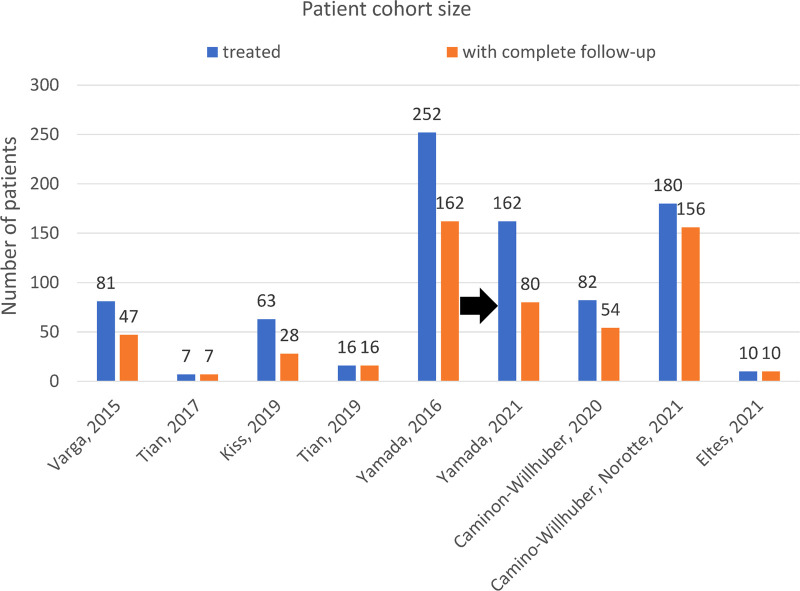
Patient involved in the clinical follow-up studies, from the initial recruitment to the final group. The difference between the number of treated patients and patients finally enrolled in the reported follow-ups was explained by the elimination of patient having an incomplete follow-up, undergoing other spine surgery following PCD, or not matching the inclusion criterion.

The first patient outcome published paper included 47 participants with complete follow-ups out of 81 initially treated patients (9). 28 participants were included by Kiss et al. in a follow-up of six months (Figure 2). The first study of Yamada et al. enrolled 162 participants (17), but was extended in a second paper, resulting in a shorter cohort of 80 participants with a complete follow-up >24 months (18). Tian et al. presented a first study gathering seven patients and a second publication with a 16 patients cohort (25, 26). Camino-Willhuber et al. presented a retrospective study on 54 participants separated into two groups: 37 participants had a degenerative scoliosis, and 17 participants did not present any sign of scoliosis (22). In a second paper, they gathered data of 156 patients from two centres that were separated into three groups based on their previous surgical history (PCD only/ PCD after previous lumbar surgery/PCD + decompression) (24).

Among the patients treated by PCD in each paper, the follow-up final participants were filtrated using exclusion criteria similar for most papers. The main exclusion criteria were:

- The absence of complete datasets (16, 22)
- The simultaneous performance of any type of spine surgery even out of L1–5 (16, 22)
- The presence of any previous surgery at the same anatomical level (17, 18, 22)

Additionally, patients with less than 1 year (22) and 2 years (24) of follow-up were excluded from Camino-Willhuber’s papers.

In order to study the impact of PCD on degenerative scoliosis, patients with a Cobb angle exceeding 10°, a VAS score above 50 points were selected, and Bone Marrow Edema visible on endplates were selected by Yamada et al.

#### VAS/ODI Scores

**Figure 3:**
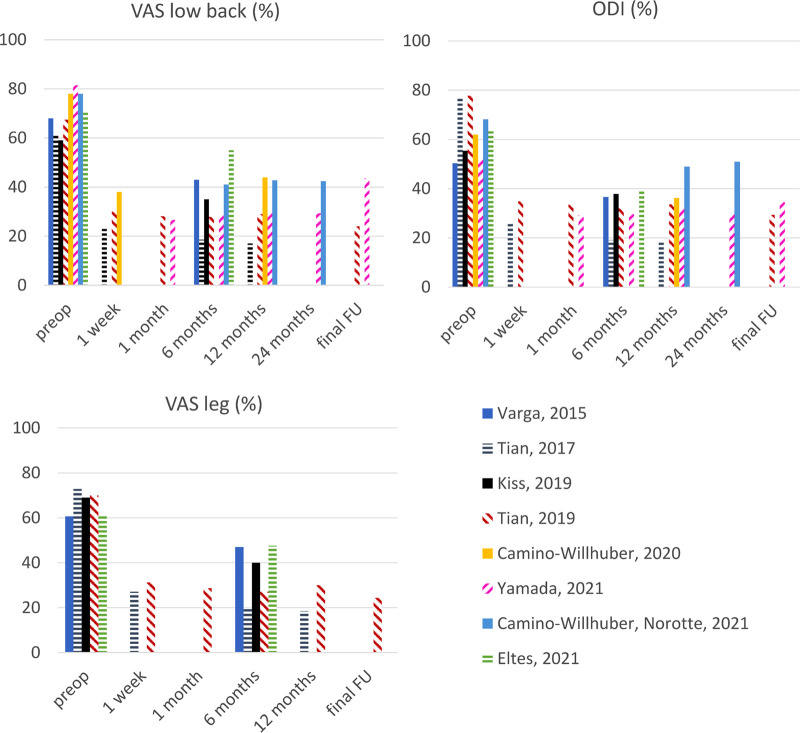
VAS and ODI scores chronologically reported from preoperative to two years postoperative. Fu: follow-up.

Low back pain graded by the VAS score was reported over the two years of follow-up (Figure 3). In all papers, the postop VAS score was significantly improved compared to preop, and at every step of the follow up. In the two longest studies, the pain level increased again with time, but remained significantly reduced compared to preoperative condition. Papers reported the disability to perform daily activities following ODI variations. Similar to VAS, all papers reported a significantly reduced ODI post-surgery compared to preoperative which was still present after two years.

#### Radiographic Parameters

Bone Marrow Edema (BME) is an accumulation of fluid in the bone marrow which can occur in case of injury or pathological condition and is associated to low back pain. Yamada et al. found a moderate positive correlation between BME and VAS as well as a weak positive correlation with ODI. The BME score decreased after PCD and for the duration of the follow up (>2 years) assessing the recession of the edema in the vertebral bodies (18).

**Figure 4:**
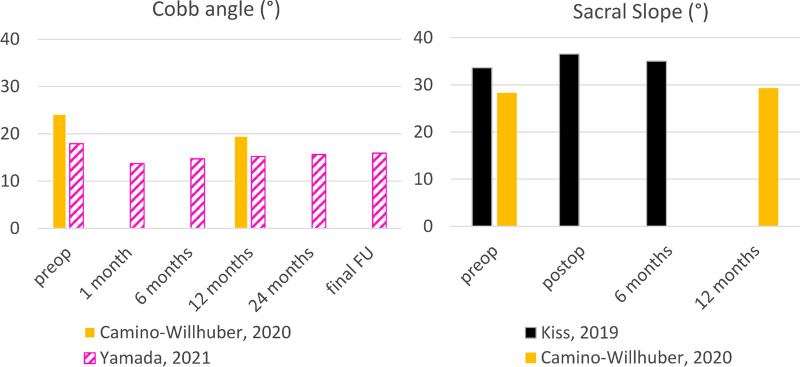
Impact of discoplasty on the sacral slope and Cobb angle.

The Cobb’s angle was measured by Yamada et al. and Camino-Willhuber et al. preop and followed for 2 years (Figure 4). After the intervention, the Cobb’s angle was significantly reduced in the scoliotic group (*p* = 0.0006), while the non-scoliotic group did not exhibit any significant change (22). The comparison between patients treated with PCD and physiologic treatments during the follow-up showed the increasing significant effect of the surgical treatment on the Cobb’s angle, however the Cobb’s angle increased during the follow-up. L1–L5 lumbar lordosis was not significantly impacted by PCD (*p* > 0.05), while the segmental (in the treated and non-treated motion segments) lordosis exhibited a significant increase (*p* < 0.05) (16). Conversely, another paper reported a significant increase of lumbar lordosis at one year postop (*p* = 0.0001) in patient with lumbar scoliosis but no significant changes in segmental lordosis (22).

The pelvic incidence remained unchanged six months after PCD (*p* > 0.05) (16). The sacral slope significantly increased postop in two papers (*p* < 0.01) and the change was maintained at follow-up (16) (Figure 4). The correction of sacral slope was positively correlated with the improvement of ODI. The pelvic tilt significantly decreased immediately after the intervention (*p* < 0.05), and the drop remained constant after 6 months (16). Lumbar lordosis was not significantly impacted by PCD (*p* > 0.05), while the segmental lordosis exhibited a significant increase (*p* < 0.05) (16). Conversely, another paper reported a significant recovery of lumbar lordosis at one year postop (*p* = 0.0001) in patients with lumbar scoliosis but no significant changes in segmental lordosis (22).

L1–L5 lumbar scoliosis and segmental scoliosis were significantly reduced in case of single-level PCD (16). The intervention significantly reduced the scoliosis angle postop (*p* < 0.05), and after 6 months no change from postop was observed (*p* > 0.05). The impact of multilevel PCD on scoliosis significantly differed from the single-level surgery: lumbar scoliosis was reduced while segmental scoliosis significantly increased.

In the sagittal plane, anterior and posterior disc height were significantly improved by PCD (*p* < 0.001 for both). The interpedicular height showed a significant increase after surgery in treated segments (*p* < 0.001) and the change was constant overtime (16).

### Biomechanical Assessment of the Effects of Discoplasty

In parallel to patient outcome investigation, *in vitro* and *in silico* studies investigated the biomechanical consequences of PCD. As application of PCD on the low thoracic/lumbar spine is recent, engineering research on the topic is currently limited.

#### Geometric Changes Associated with PCD

The first interest of the technical papers was to provide objective data to evaluate the success of the surgery to match the clinical expectations. In order to relieve pain, PCD aimed to fill VP with acrylic bone cement in order to increase the disc height and achieve an increase of the foramen space.

Postoperatively, the *in vivo* cement distribution was segmented from CT scans and characterised in terms of volume and surface of the cement mass by Eltes et al. (21). The cement axial thickness between the endplates was also measured for each treated disc. A large variability of volume (3.8–13.1 mL range) and shape was reported, which was induced by the wide variations of musculoskeletal status and degeneration of each patient. Improvement of the patient outcome was correlated to thicker cement mass. In addition, in an *in vitro* study written by our group and currently in submission, discoplasty was reproduced on 27 cadaveric specimens and the volume of injected cement was measured on CT scans images. Supporting Eltes’ conclusions, the volume of cement varied widely between specimens (2.0–8.9 mL range) within the same range as *in vivo* measurements.

Techens et al. compared ten porcine lumbar discs *in vitro*, in the intact condition, after nucleotomy, and after simulated PCD tested in flexion and extension. In both motions, the posterior disc height decreased by more than 15% after nucleotomy, whereas discoplasty significantly restored it. In extension, the posterior disc height after surgery did not differ significantly from the intact disc. This *in vitro* investigation confirmed the disc height increase clinically observed (28). The same protocol was applied to 27 cadaveric specimens (Techens et al., submitted) by our group and PCD significantly increased posterior disc height in flexion (41% ± 46%) and extension (35% ± 38%) in comparison to after nucleotomy.

Eltes et al. developed an 3D volumetric method to quantify the preop-postop change of the foramen space from tomographic images. PCD significantly decompressed the foramens despite the wide difference of volumetric changes (mean = 2295 mm^3^, SD = 1181, *n* = 16). Foraminal decompression was favoured by higher volume, larger surface and lower surface-volume ratio (21).

#### Biomechanical Properties of the Spine After Discoplasty

Although PCD does not primarily aim to stabilize the spine, stability is often an additional concern in disc degeneration. Techens et al. measured the *in vitro* range of motion and stiffness following PCD on porcine lumbar segments (28). No significant change was observed despite a decrease of the ROM in flexion and an increase in extension compared to intact discs. Discoplasty recovered the intact ROM compared to nucleotomy. The strains measured after discoplasty on the specimen surface partially regained the distribution observed with intact discs. PCD also reduced the peak strains observed after nucleotomy. Another study under submission investigated the *in vitro* range of motion and stiffness following PCD on human lumbar segments. PCD significantly reduced the ROM and increased the elastic stiffness in flexion only. In addition, the laxity zone was significantly shortened by the surgery in both motions. The strain intensity measured on the specimen disc surface decreased after PCD compared to the distribution in nucleotomy. Besides, in both motions the specimens exhibited lower peak strain values after the surgery, indicating no local extreme tissue deformation.

### Alternative Materials for Discoplasty

Research on PCD also covered improvements of the technique to provide a better stabilization of the spine and improvement of patient’s condition. Osteogenic mineralized collagen (MC) modified polymethylmethacrylate (PMMA) cement was investigated by Yang et al. as a substitute to acrylic bone cement for PCD. With MC particle size ranging between 300 and 400 micrometers, injectability, hydrophilicity, and mechanical properties of MC-PMMA were characterised (29). After implantation in goat, MC-PMMA showed a significant better osteointegration than standard acrylic cement with a higher ratio between the cement surface in contact with bone and the cement total surface (circumferential contact index). Moreover, MC-PMMA triggered a limited reaction from the immune system, in comparison to standard acrylic cement which exhibited a large fibrous encapsulation. MC-modified PMMA exhibited significantly reduced stiffness (three-points bending elastic modulus of 2.4–2.8 GPa for frequencies of 1–10 Hz), which supposedly would reduce the risks of bone fracture. Thus, MC-PMMA was presented as a promising alternative to pure acrylic bone cement for disc degeneration treatment with PCD. Targeting the same objective of injecting a material which would reduce mechanical stresses on the endplates, Lewin et al. developed an *in silico* model of the spine in order to test low modulus PMMA cements (27). Three modified PMMA-based cements with different concentrations of linoleic acid (LA) were tested *in vitro* to extract mechanical parameters. Elastic modulus of LA-PMMA was up to ten times smaller than the original PMMA-based cement, however the modulus increased over time. The numerical model showed that the stress average increased on the endplates after discoplasty, but the stresses decreased with higher content of LA. This material seems also a promising alternative to acrylic cement for discoplasty although some aspects still require optimization, such as the material mechanical stability over time.

### Limitations and Risks of Discoplasty

As PCD is a minimally invasive surgical technique, it reduces the risks of clinical complications compared to the open surgical treatments of degenerative disc disease. However, it still implies limitations and risks reported by the previous papers. Among the rare permanent complications reported, cement leakage in the intervertebral foramen and vertebral body fracture were the most common (<5% and <1% respectively) (16, 18, 24). Unlike leakage in the adjacent vertebrae which are harmless, cement in the intervertebral foramen could jeopardize the spinal cord integrity. The incidence can be limited by closely monitoring cement injection with fluoroscopy, and using intraoperative neurophysiological monitoring to adapt the approach, entry point and direction chosen for the injection (see 3.2.4).

Vertebral fractures are naturally prevented by endplate sclerosis, and by selecting patients with sufficient bone density. Pre-operative treatments can also be implemented to strengthen the bone structures. Additionally, PCD creates a patient-specific cement spacer adapted to the endplate shape: this increases the contact surface for the transmission of the loads at the cement-endplate interface. Although no dedicated investigation of the intra-discal stress and subsidence after PCD has been conducted so far, an increased bearing surface can be expected to reduce the pression on the endplates compared to other non-specific devices previously used to space the vertebrae (36). Finally, vertebral fractures could be prevented by replacing the injected bone cement with substitute fillers exhibiting reduced mechanical stiffness.

Other concerns can be raised about the interface between the cement and the surrounding annulus. No paper could be found focusing on both the short- and long-term *in vivo* responses of the biological tissue of the disc to the presence of the injected cement. No abnormal inflammatory activity was reported in the follow-ups. Acrylic cement being biocompatible and favouring osteointegration, long-term cemented discs would be expected to fuse and stabilize the treated level. Complications arising from long-term motion such as cement loosening or wear although they have not been studied yet, would therefore seem unlikely. However, substitute filler with better osteointegration would still decrease these risks of complications.

## Discussion and Conclusions

Disc degeneration disease has a high prevalence, particularly in the elderly, and is responsible for low back pain (1). In the most severe cases, the disappearance of the nucleus pulposus results in the presence of a vacuum which leads the disc to collapse, thus reducing the clearance of the foramens. As polymorbid and old patients are not eligible for an open surgery, they are sometimes treated with a minimally invasive surgery, percutaneous cement discoplasty (9). The aim of this review was to establish a state-of-the-art of the publications related to PCD.

Twenty papers were retrieved through two databases covering clinical and engineering approaches of the surgery. Two papers presented the operative technique and described the criteria for patient selection (9, 19). PCD consists in filling the intradiscal space with injectable acrylic bone cement to replace the VP by a cemented spacer. Patients are usually discharged between one and three days after surgery. PCD is mainly contraindicated in case of severe osteoporosis and severe spine deformity although it stabilized degenerative scoliosis (19). Cement leakage in the vertebral bodies or the foramen are the most common complications but in all reported cases, it had a low prevalence (4% of the treated discs).

Nine papers reported clinical follow-up lasting between 6 months and 4 years for 28–162 patients (9, 16–18, 21, 22, 24–26). All follow-ups concluded that PCD significantly reduced low back pain immediately after surgery and that pain was still relieved at the end of the follow-up. Similarly, the quality of life reported by patient significantly improved post-surgery and the improvement lasted until the end of the study. Patient outcome correlated with the increase of the foramen space following the surgery (21). The disc height was restored by PCD, validating the main objective of surgery. PCD significantly impacted some radiographic parameters, among which the scoliosis angle although the surgery is not primarily recommended to treat scoliosis. Biomechanical studies showed that PCD restored the spine stability during flexion and extension and did not induce irregular deformation of the surface disc tissue (28).

Among the investigations on PCD, two research papers presented variations of bone cement (MC-PMMA and LA-PMMA) as filling material (27, 29). MC-PMMA exhibited a better osteointegration and triggered less the immune system reaction compared to pure acrylic cement, and LA-PMMA reduced the stresses on the endplates reducing risk of bone marrow edema.

The literature reviewed seems to show that PCD is a safe and effective MIS procedure for the treatment of advanced stage disc degeneration in selected cases. However, studies comparing the effectiveness of PCD to conventional treatment options were unavailable. The review showed a major limitation of the clinical studies: only static supine and standing position (loaded by the upper body weight) was investigated. However, axial compression is not the only challenge for disc height and foramen space. The study of potential damaging activities or spine motions was omitted. Questions such as: “Which load could a patient safely carry? Which movement could be safely performed?” were not investigated yet, although patients indicated for PCD were unlikely to carry heavy loads or ostentatiously exercise. Additionally, the spine biomechanical behaviour under various loadings just started to be studied. Thus, a focus on other loading configurations as well as the measurement of different parameters would be needed to complete a rational on the benefits and limitations of percutaneous cement discoplasty.

Directions for possible future research in this area include alternative injectable materials for better biomechanical and clinical performance. Clinical and biomechanical investigations would help optimizing the surgical technique, including point of needle insertion and of cement delivery. Also, one should remember the frame of application of PCD and conduct more investigations in case of change of the indications of the surgery (younger, more active patients, etc.).

## Data Availability

The datasets presented in this study can be found in online repositories. The names of the repository/repositories and accession number(s) can be found below: Figshare folder, DOI: 10.6084/m9.figshare.19375604.

## References

[1] Wang Y Battié MC. Epidemiology of lumbar disc degeneration. In: Shapiro IM Risbud MV, editors. The intervertebral disc: Molecular and structural studies of the disc in health and disease. Vienna: Springer (2014). p. 139–56. 10.1007/978-3-7091-1535-0_9

[2] Vergroesen P-PA Kingma I Emanuel KS Hoogendoorn RJW Welting TJ van Royen BJ Mechanics and biology in intervertebral disc degeneration: a vicious circle. Osteoarthr Cartil. (2015) 23(7):1057–70. 10.1016/j.joca.2015.03.028 25827971

[3] Lee SY Kim T-H Oh JK Lee SJ Park MS. Lumbar stenosis: a recent update by review of literature. Asian Spine J. (2015) 9(5):818–28. 10.4184/asj.2015.9.5.818 26435805 PMC4591458

[4] Morishita K Kasai Y Uchida A. Clinical symptoms of patients with intervertebral vacuum phenomenon. Neurologist. (2008) 14(1):37–9. 10.1097/NRL.0b013e3180dc9992 18195655

[5] Wu PH Kim HS Jang I-T. Intervertebral disc diseases PART 2: a review of the current diagnostic and treatment strategies for intervertebral disc disease. Int J Mol Sci. (2020) 21(6):2135. 10.3390/ijms21062135 PMC7139690 32244936

[6] Fernandez-Moure J Moore CA Kim K Karim A Smith K Barbosa Z Novel therapeutic strategies for degenerative disc disease: Review of cell biology and intervertebral disc cell therapy. SAGE Open Med. (2018) 6:2050312118761674. 10.1177/2050312118761674 29568524 PMC5858682

[7] Stergar J Gradisnik L Velnar T Maver U. Intervertebral disc tissue engineering: A brief review. Bosnian J Basic Med Sci. (2019) 19(2):130–7. 10.17305/bjbms.2019.3778 PMC6535390 30726701

[8] Wang Y Bai Y Ma H Wang S. Comparison of total disc arthroplasty and fusion in treatment of lumbar disc disease. Medicine. (2020) 99(35):e22024. 10.1097/MD.0000000000022024 32871957 PMC7458242

[9] Varga PP Jakab G Bors IB Lazary A Szövérfi Z. Experiences with PMMA cement as a stand-alone intervertebral spacer: Percutaneous cement discoplasty in the case of vacuum phenomenon within lumbar intervertebral discs. Der Orthopade. (2015) 44(Suppl 1):S1–S7. 10.1007/s00132-014-3060-1 25875227

[10] Roosen K. Bone cement as replacement material of cervical disks. Fortschr Med. (1982) 100(45):2120–6. 7173797

[11] Bärlocher CB Barth A Krauss JK Binggeli R Seiler RW. Comparative evaluation of microdiscectomy only, autograft fusion, polymethylmethacrylate interposition, and threaded titanium cage fusion for treatment of single-level cervical disc disease: a prospective randomized study in 125 patients. Neurosurg Focus. (2002) 12(1):1–7. 10.3171/foc.2002.12.1.5 16212331

[12] Korinth MC Krüger A Oertel MF Gilsbach JM. Posterior foraminotomy or anterior discectomy with polymethyl methacrylate interbody stabilization for cervical soft disc disease: results in 292 patients with monoradiculopathy. Spine. (2006) 31(11):1207–14; discussion 1215–6. 10.1097/01.brs.0000217604.02663.59 16688033

[13] Kettler A Wilke H-J Claes L. Effects of neck movements on stability and subsidence in cervical interbody fusion: an in vitro study. J Neurosurg. (2001) 94(1):97–107. 10.3171/spi.2001.94.1.0097 11147875

[14] Wilke HJ Kettler A Goetz C Claes L. Subsidence resulting from simulated postoperative neck movements: an in vitro investigation with a new cervical fusion cage. Spine. (2000) 25(21):2762–70. 10.1097/00007632-200011010-00008 11064521

[15] Wilke H-J Kettler A Claes L. Primary stabilizing effect of interbody fusion devices for the cervical spine: an in vitro comparison between three different cage types and bone cement. Eur Spine J. (2000) 9(5):410–6. 10.1007/s005860000168 11057535 PMC3611385

[16] Kiss L Pal Varga P Szoverfi Z Jakab G Eltes PE Lazary A. Indirect foraminal decompression and improvement in the lumbar alignment after percutaneous cement discoplasty. Eur Spine J [Preprint]. (2019) 28:1441–7. 10.1007/s00586-019-05966-7 31006068

[17] Yamada K Nakamae T Shimbo T Kanazawa T Okuda T Takata H Targeted therapy for low back pain in elderly degenerative lumbar scoliosis: a cohort study. Spine. (2016) 41(10):872–9. 10.1097/BRS.0000000000001524 26909842

[18] Yamada K Nakamae T Nakanishi K Kamei N Hiramatsu T Okuda T Long-term outcome of targeted therapy for low back pain in elderly degenerative lumbar scoliosis. Eur Spine J. (2021) 30:2020–32. 10.1007/s00586-021-06805-4 33733329

[19] Sola C Percutaneous cement discoplasty for the treatment of advanced degenerative disk disease in elderly patients. Eur Spine J. (2021) 30:2200–8. 10.1007/s00586-018-5547-7 29569159

[20] Camino-Willhuber G Mariana Bendersky M De Cicco FL Kido G Duarte MP Estefan M Development of a new therapy-oriented classification of intervertebral vacuum phenomenon with evaluation of intra- and interobserver reliabilities. Global Spine J. (2021) 11(4):480–7. 10.1177/2192568220913006 32875883 PMC8119922

[21] Eltes PE Laszlo Kiss L Bereczki F Szoverfi Z Techens C Jakab G A novel three-dimensional volumetric method to measure indirect decompression after percutaneous cement discoplasty. J Orthop Translat. (2021) 28:131–9. 10.1016/j.jot.2021.02.003 33898249 PMC8050383

[22] Camino-Willhuber G Kido G Duarte MP Estefan M Bendersky M Bassani J Percutaneous cement discoplasty for the treatment of advanced degenerative disc conditions: a case series analysis. Global Spine J. (2020) 10(6):729–34. 10.1177/2192568219873885 32707012 PMC7383797

[23] Camino-Willhuber G Bendersky M Vilte C Kido G Duarte MP Estefan M Accuracy of intraoperative neuromonitoring during percutaneous cement discoplasty. Rev Fac Cienc Med. (2021) 78(3):257–63. 10.3105310.31053/1853.0605.v78.n3.32619 PMC8760906 34617703

[24] Camino-Willhuber G Norotte G Bronsard N Kido G Pereira-Duarte M Estefan M Percutaneous cement discoplasty for degenerative low back pain with vacuum phenomenon: a multicentric study with a minimum of 2 years of follow-up. World Neurosurg. (2021) 155:e210–e217. 10.1016/j.wneu.2021.08.042 34403794

[25] Tian Q-H Lu Y-Y Sun X-Q Wang T Wu C-G Li M-H Cheng-Ying S. Feasibility of percutaneous lumbar discectomy combined with percutaneous cementoplasty for symptomatic lumbar disc herniation with modic Type I endplate changes. Pain Physician. (2017) 20(4):E481–8. PMID: 28535556

[26] Tian Q-H Liu Z-J Liu H-F Fang R Shen R-R Wang T Cheng Y-S Safety and efficacy of percutaneous lumbar discectomy and percutaneous disc cementoplasty for painful lumbar disc herniation in patients over 60 years. J Vasc Interv Radiol. (2019) 30(6):894–9. 10.1016/j.jvir.2018.12.018 30952522

[27] Lewin S Försth P Persson C. Low-modulus PMMA has the potential to reduce stresses on endplates after cement discoplasty. J Funct Biomater. (2022) 13(1):18. 10.3390/jfb13010018 35225981 PMC8883899

[28] Techens C Palanca M Éltes PE Lazáry Á Cristofolini L. Testing the impact of discoplasty on the biomechanics of the intervertebral disc with simulated degeneration: An in vitro study. Med Eng Phys. (2020) 84:51–9. 10.1016/j.medengphy.2020.07.024 32977922

[29] Yang L Kong J Qiu Z Shang T Chen S Zhao R Raucci MG Mineralized collagen-modified PMMA cement enhances bone integration and reduces fibrous encapsulation in the treatment of lumbar degenerative disc disease. Regen Biomater. (2020) 7(2):181–93. 10.1093/rb/rbz044 32296537 PMC7147368

[30] Wang B Shan L Hao D. Letter to the Editor concerning “Percutaneous cement discoplasty for the treatment of advanced degenerative disk disease in elderly patients” by Sola C, Camino Willhuber G, Kido G et al. Eur Spine J (2018): Doi 10.1007/s00586-018-5547-7. Eur Spine J. (2018) 27(7):1665–6. 10.1007/s00586-018-5642-9 29802466

[31] Camino-Willhuber G Sola C. Answer to the Letter to the Editor of Biao Wang et al. concerning “Percutaneous cement discoplasty for the treatment of advanced degenerative disk disease in elderly patients” by Sola C, Camino Willhuber G, Kido G et al. Eur Spine J (2018): doi: 10.1007/s00586-018-5547-7. Eur Spine J. (2018) 27(7):1667–8. 10.1007/s00586-018-5643-8 29804182

[32] Lazary A. Expert’s Comment concerning Grand Rounds Case entitled “Percutaneous cement discoplasty for the treatment of advanced degenerative disk disease in elderly patients”. Eur Spine J. (2021) 30(8):2209–10. 10.1007/s00586-020-06568-4 32813038

[33] Camino-Willhuber G Bendersky M Bianchi H Gruenberg M Sola C. Reply to A. Lazary’s Expert’s Comment concerning grand rounds case entitled “Percutaneous cement discoplasty for the treatment of advanced degenerative disk disease in elderly patients” (C. Sola, et al., Eur Spine J; 2021;30(8):2209–2210. doi: 10.1007/s00586-020–06568-4). Eur Spine J. (2022) 31(1):205–6. 10.1007/s00586-021-06988-w 34495392

[34] Lee GY Lee JW Choi HS Oh K-J Kang H-S. A new grading system of lumbar central canal stenosis on MRI: an easy and reliable method. Skelet Radiol. (2011) 40(8):1033–9. 10.1007/s00256-011-1102-x 21286714

[35] Bartynski WS Lin L. Lumbar root compression in the lateral recess: MR imaging, conventional myelography, and CT myelography comparison with surgical confirmation. AJNR Am J Neuroradiol. (2003) 24(3):348–60. PMID: ; PMCID: 12637281 PMC7973614

[36] Patel RR. *Does patient-specific implant design reduce subsidence risk in lumbar interbody fusion? A bottom up analysis of methods to reduce vertebral endplate stress*. Ph.D. (2018). Available from: https://www.proquest.com/docview/2198656190/abstract/FF7B04C784894A33PQ/1 (Accessed February 3, 2022).

